# Underlying molecular and cellular mechanisms in childhood irritable bowel syndrome

**DOI:** 10.1186/s40348-016-0036-8

**Published:** 2016-02-16

**Authors:** Bruno P. Chumpitazi, Robert J. Shulman

**Affiliations:** Department of Pediatrics, Baylor College of Medicine, One Baylor Plaza, Houston, 77030 TX USA; Gastroenterology, Hepatology, and Nutrition, Texas Children’s Hospital, 6621 Fannin Street, 77030 Houston, TX USA; Children’s Nutrition Research Center, 1100 Bates Avenue, Houston, 77030 TX USA

**Keywords:** Irritable bowel syndrome, Children, FODMAP, Bile acid, Serotonin, Genetics

## Abstract

Irritable bowel syndrome (IBS) affects a large number of children throughout the world. The symptom expression of IBS is heterogeneous, and several factors which may be interrelated within the IBS biopsychosocial model play a role. These factors include visceral hyperalgesia, intestinal permeability, gut microbiota, psychosocial distress, gut inflammation, bile acids, food intolerance, colonic bacterial fermentation, and genetics. The molecular and cellular mechanisms of these factors are being actively investigated. In this mini-review, we present updates of these mechanisms and, where possible, relate the findings to childhood IBS. Mechanistic elucidation may lead to the identification of biomarkers as well as personalized childhood IBS therapies.

## Introduction

Chronic abdominal pain is highly prevalent affecting up to 25 % of schoolchildren worldwide [1, 2]. Using conventional diagnostic tools, the majority of children with chronic abdominal pain will not have an organic etiology; as a result, they are diagnosed as having an abdominal pain-related functional gastrointestinal disorder (FGID) [3, 4].

Based on the symptoms suffered by the individual, FGIDs are classified further into functional dyspepsia, functional abdominal pain, and irritable bowel syndrome (IBS) [3]. Of the abdominal pain-related FGIDs, IBS is the most prevalent, encompassing approximately 60 % of children with a FGID [5]. Based on the predominant stool form, IBS is further phenotypically subtyped into IBS with constipation (IBS-C), IBS with diarrhea (IBS-D), IBS mixed subtype (IBS-M), and IBS un-subtyped (IBS-U) [6]. These subtypes occur in children and adults with IBS; however, in children IBS-C and IBS-U are more prevalent than in adults [7]. It is generally believed that IBS subtyping increases the likelihood of identifying underlying unique pathobiological processes specific to each IBS subtype [6]. However, it should be noted that IBS subtype may change over 12 months in up to 24 % of children [8].

**Fig. 1:**
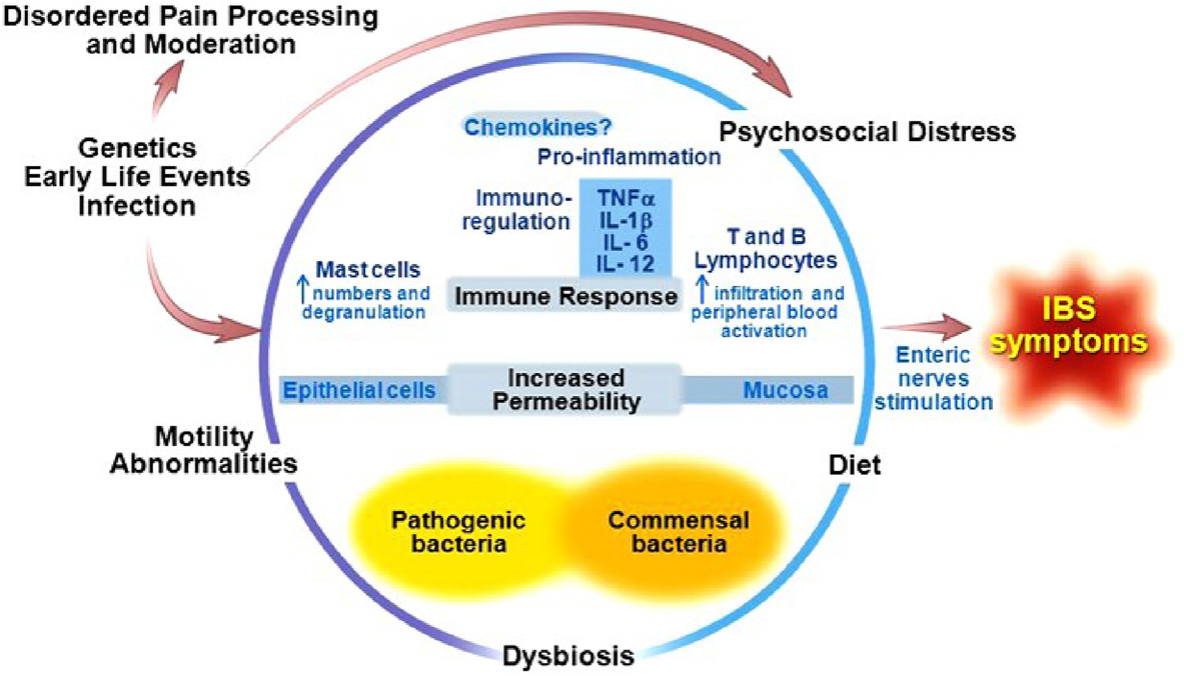
The biopsychosocial model of irritable bowel syndrome. The *circle* represents the interrelated nature of the model. Several factors (in *black bold*) are present and affect other factors as shown by the direction of the *red arrows*. Mechanisms related to these factors are in *blue font* within the *circle*. The balance between pathogenic bacteria and commensal bacteria is in *yellow*. These factors ultimately come together and lead to IBS symptoms. Figure is modified from Rodriguez-Fandino et al. [10] and reproduced by permission of Journal of Neurogastroenterology and Motility

There are several factors which may play a role in the symptom expression in a child with IBS including: visceral hyperalgesia, intestinal hyperpermeability, gut microbiome composition, psychosocial distress, food intolerance, colonic bacterial fermentation, genetics, and gut inflammation [5, 9]. Each process may play a lesser or greater role in one individual as compared to another; therefore, IBS is often approached via a biopsychosocial model (Fig. 1) [10].

Investigators continue to actively explore several concurrent areas of potential IBS pathobiology. In this mini-review, we present updates within several potential pathobiological factors in IBS with an emphasis on the underlying molecular and cellular mechanisms. When possible, we focus on data and studies relevant specifically to children. Further understanding cellular and molecular mechanisms may one day lead to both the identification of reliable biomarkers as well as personalized IBS therapies.

### Visceral hypersensitivity

Visceral sensation mechanisms and pathways have been extensively reviewed by others [11]. Communication of sensory nerves responsive to noxious stimuli (chemical, mechanical, or inflammatory) with the central nervous system occurs via vagal, pelvic, and splanchnic nerve pathways [11, 12]. Splanchnic and pelvic nerve signals are transmitted via spinal visceral afferents to the spinal dorsal horn where they subsequently communicate to the brain via several ascending pathways including the spinothalamic, spinoreticular, and spinomesencephalic tracts. Descending inhibitory pathways influence visceral sensation by modulating (gating) the ascending visceral afferent pathways [12]. Visceral sensation processing is further modulated by mediators and receptors including neurotransmitter receptors, cannabinoid receptors, opioid receptors, gamma-aminobutyric acid receptors, glutamate receptors, glucocorticoid receptors, inflammatory receptors, and ion channel receptors [11]. Additional psychological factors such as attention/distraction, expectations of pain, emotion, stress, and coping strategies also play a role (Fig. 1).

Visceral hypersensitivity may be present in a subgroup of children with IBS. Through the use of rectal barostat studies, which inflate a balloon in the rectum while measuring pressure and volume, some children with IBS have been found to have increased visceral sensitivity [13]. In a small rectal barostat study in children with IBS (*n* = 10), Iovino et al. identified that emotional instability (anxiety, depression, discontent, impulsiveness, and anger) modulated visceral perception [14]. Supporting this are data suggesting children with pain-related FGIDs who had more anxiety and somatization also had increased pain frequency and severity; however, the study did not measure visceral sensitivity [15].

Abnormalities within the descending pain modulatory system are increasingly considered to be important in the development of the pro-nociceptive state encountered with visceral hypersensitivity [12]. Williams et al. recently found, using a diffuse noxious inhibitory control study design, that girls with IBS (in comparison to healthy girls) had impaired pain inhibition [16]. Hypnotherapy (hypnosis with therapeutic intent) appears, in part, to use descending inhibitory pathways for control of pain [17] while also reducing psychological factors such as stress [18]; it has demonstrated efficacy in several randomized controlled trials for childhood IBS [19].

While the majority of work in new pharmacological visceral pain therapies is occurring within animal models, there are new emerging clinical therapies. For example, efforts have focused on inflammatory receptors known as tachykinin receptors, of which there are three types [20]. Within a phase II randomized, placebo-controlled, parallel group study, Ibodutant^®^ (MEN 15596), a neurokinin 2 antagonist, demonstrated outcome efficacy (overall IBS symptom relief for 3 of 4 weeks) in a post hoc analysis of adults with IBS-D with pain [20]. However, it should be noted that in the overall IBS population studied, Ibodutant^®^ was not superior to placebo. In infants with colic, an abstract has reported that Nepadutant^®^ (MEN-11420), a neurokinin 2 antagonist, had efficacy in a phase II, randomized, double blind, placebo-controlled trial [21]. Though infants with colic may go on to manifest childhood FGIDs [22], there are currently no studies using neurokinin 2 antagonists in childhood IBS. We anticipate further studies with new pharmacologic therapies directed at modulating visceral pain.

In addition to drug development, alternative therapies are now being investigated for their ability to modulate visceral pain. Menthol, an active ingredient of peppermint oil, may reduce visceral pain through ion channels such as transient receptor potential ion channel melastatin subtype 8 [23]. A recent meta-analysis of peppermint oil identified an improvement in global assessment and IBS scores in adults with IBS [24]. Given a previous successful small, short-term study in children with IBS [25] and a recent small pharmacodynamic study in children with IBS [26], we anticipate further studies of peppermint oil in children with IBS.

### Genetic factors and serotonin signaling

Genetic factors in IBS recently have been reviewed in detail and are beyond the scope of this review [27]. Therefore, we provide a brief overview with more focus on genetics as they relate to serotonin signaling.

Relatives with a family history of IBS have a two- to threefold increased risk for IBS [27]. Twin studies have estimated the genetic heritability of IBS to range between 22 and 57 % [28] with higher prevalence seen amongst monozygotic in comparison to dizygotic twins [29]. The potential role of environmental contributions is still being investigated.

Several genetic disturbances related to IBS have been identified [27]. Perhaps the most extensive investigations to date have related to 5-hydroxytryptamine (5-HT, serotonin). Serotonin is a bioamine neurotransmitter primarily produced in the gut which is important for modulating intestinal motility, inflammation, and secretion [30]. Serotonin is synthesized by tryptophan hydroxylase within enteroendocrine cells and enteric neurons [31]. Under normal circumstances, the serotonin transporter (SERT) from gene SLC6A4 terminates 5-HT action via uptake into enterocytes and serotonergic neurons. However, genotypes which would alter SERT expression have been identified. These include homozygous genotypes of the promoter length polymorphism 5-HT transporter-linked polymorphic region (5-HTTLPR) which is upstream of SLC6A4. Short allele polymorphisms affect SLC6A4 transporter expression and have been associated with IBS-D and IBS-C [27]. 5-HT levels have been found to be markedly increased in rectal biopsies of adults with IBS-D who were homozygous for the short allele for 5-HTTLPR [32]. However, the field is still being actively investigated as a recent meta-analysis identified that the long/long genotype of 5-HTTLPR is associated with IBS-C but did not find associations of 5-HTTLPR mutations with IBS-D [33].

Beyond the gut alone, the short allele of 5-HTTLPR also is associated with several other factors including depression, anxiety, neuroticism, anxiety, increased sympathetic tone, decreased parasympathetic tone, post-infectious IBS, and higher cortisol levels [27]. Whether this and/or other serotonin-related mutations are found in children with IBS is currently unknown.

### Altered intestinal permeability

Intestinal permeability is regulated, in part, by inter-epithelial tight junctions composed of protein complexes. Within these protein complexes, three proteins have received particular attention within IBS: occludin, claudin-1, and zonula occludens-1 [34]. Though these protein complexes were not specifically evaluated, increased intestinal permeability has been identified in children with IBS [35]. In adults with IBS, increased intestinal permeability correlates with increased visceral hypersensitivity and abdominal pain symptoms [36].

Intestinal permeability recently has been found to be regulated by a class of endogenously expressed noncoding RNAs (21–23 nucleotides in length) known as microRNAs. MicroRNAs regulate gene expression, including gene expression of tight junction proteins. Specifically, in adults with IBS-D, by downregulating nuclear factor-ĸβ-repressing factor and claudin-1 mRNA expression, microRNA 29 increases intestinal barrier permeability [37]. Further studies to understand the unique role of microRNA 29 in those with IBS-D as opposed to other subtypes are needed. In addition, it is not currently known whether these molecules may serve as biomarkers in children with IBS.

Nevertheless, within the field of gastroenterology, there are increasing efforts to address pathology by improving intestinal barrier function. In children with IBS, a randomized controlled trial demonstrated that *Lactobacillus* GG improved both abdominal pain and intestinal permeability [38]. In adults with IBS, glutamine increased claudin-1 expression in patients with IBS-D [34]. In adults with celiac disease, Leffler et al. have studied the effect of larazotide (an inhibitor of *Vibrio cholera* zonula occludens toxin) which promotes tight junction assembly [39]. Though there were mixed results, a low dose of larazotide in adults with celiac disease on a gluten-free diet improved persistent symptoms [39].

We anticipate there will be further efforts in children with IBS to address increased intestinal permeability. In addition to specific therapies directed at tight junction proteins, there is increasing knowledge of other factors that alter intestinal permeability such as stress and immune activation (Fig. 1) [40].

### Gut epithelial immune activation

#### Mast cells

**Fig. 2:**
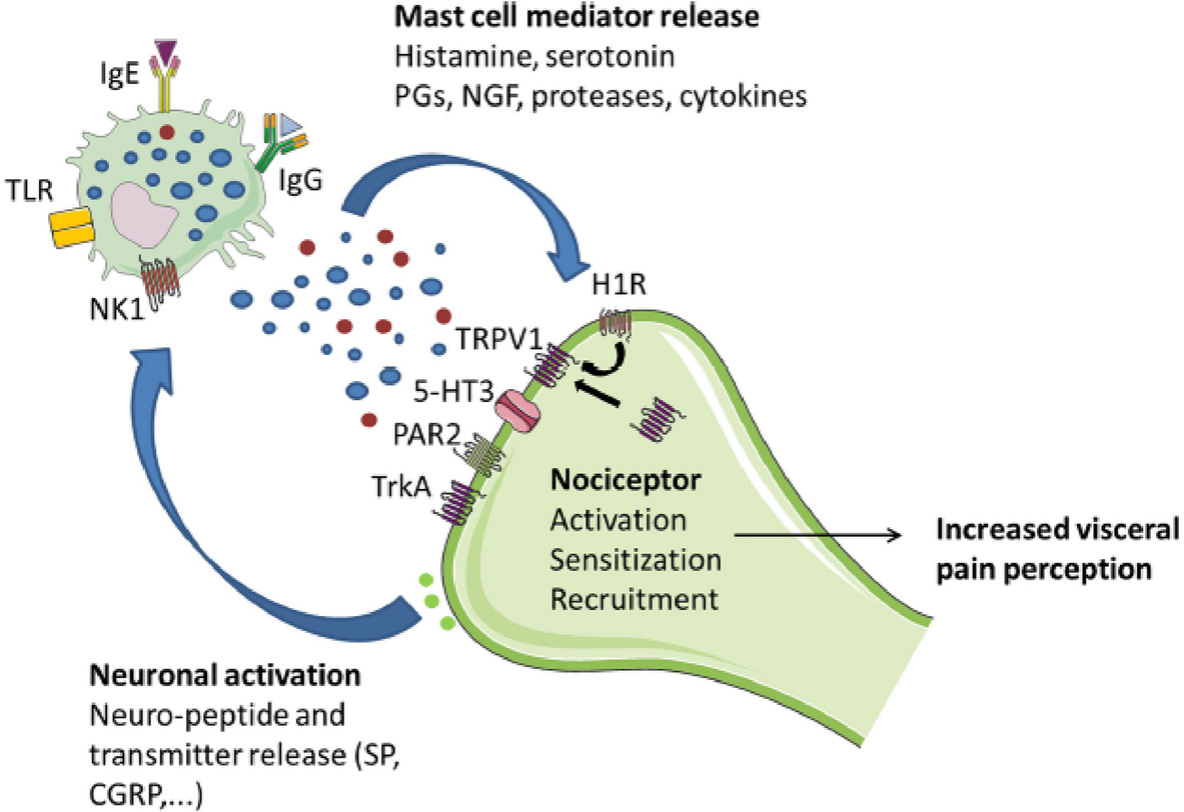
Mast cell-nerve interactions in the human gut. Mast cells and nerves communicate bidirectionally, thereby modulating peristalsis and pain signaling. The release of bioactive, pro-inflammatory mediators by mast cells results in a variety of neuronal effects including activation, sensitization, and recruitment of nociceptors to the cell membrane, neurogenic inflammation, and neural sprouting. Ultimately, this leads to visceral hypersensitivity. Neuronal activation triggers the release of neuropeptides and neurotransmitters, thereby further activating mast cells. H1R = histamine-1 receptor; TRPV1 = transient receptor vanilloid 1; 5-HT3 = 5-hydroxytryptamine receptor; PAR2 = proteinase-activated receptor-2; TrkA = receptor for nerve growth factor; TLR = toll-like receptor; NK1 = neurokinin 1 receptor; SP = substance P; CGRP = calcitonin-related gene peptide; Ig = immunoglobulins; NGF = neuronal growth factor; PG = prostaglandins. Figure reproduced from “The role of mast cells in functional GI disorders,” Wouters et al. [41] with permission from BMJ Publishing Group Ltd.^©^ 2015 BMJ & British Society of Gastroenterology. All rights reserved

Several studies in both children and adults with IBS have identified increased gut epithelial immune activation in those with FGIDs [35]. Perhaps the most compelling data regarding immune activation relate to mast cells. These cells release newly synthesized and stored mediators including cytokines, histamine, serotonin, prostaglandins, neuronal growth factor, proteases, tryptase, and other bioactive substances (Fig. 2). These mast cell mediators may cause epithelial and neuromuscular dysfunction; this in turn promotes visceral hypersensitivity, altered motility, and increased intestinal permeability [41]. Enteric nerves have receptors for the mast cell mediators which lead to nociceptor activation, sensitization, recruitment, and ultimately increased visceral hypersensitivity (Fig. 2). Triggers of mast cell release include the following: psychological stress via corticotropin-releasing factor; immunoglobulins, such as IgE; neurotransmitters, such as histamine; neuropeptides, such as substance P and calcitonin-related gene peptide (Fig. 2); growth factors, such as nerve growth factor; biological molecules, such as lipopolysaccharide; and physiochemical stimuli such as pressure [41].

In adults with IBS, though not consistently identified, mast cells in the terminal ileum and throughout the colon may be found in increased numbers [42, 43]. These mast cells may be found adjacent to nerve fibers, which lead credence to their potential pathophysiologic role [41]. In adults with IBS, the number of mast cells correlates with IBS abdominal pain symptoms [41]. Although the number of mast cells in ileocolonic biopsies did not differ between children with IBS and controls, Di Nardo et al. found that in children with IBS there was an increased number of mast cells in close proximity to nerves [44]. In addition, Di Nardo et al. found that in children with IBS, the mast cell number in ileocolonic biopsies correlated with both abdominal pain frequency and severity [44].

Therapies directed toward stabilization of mast cells have demonstrated efficacy. Cromolyn, in a small (*n* = 16) randomized crossover trial in adults with IBS, improved abdominal pain severity [45]. In a randomized, parallel, controlled trial in adults with IBS, ketotifen (a mast cell stabilizer) both decreased visceral hypersensitivity and improved intestinal symptoms [46]. However, it is important to note that many of these improvements were lost during multiple testing in the Klooker et al. trial. Though mast cell stabilizers have not been evaluated in children with IBS, a retrospective study of cyproheptadine, which has several properties including being an H_1_ antagonist, ameliorated abdominal pain in children with FGIDs including IBS [47]. Further studies evaluating mast cell therapies are needed.

#### Humoral immune activation

Adults with IBS have been shown to have humoral immune activation (Fig. 1). Adults with IBS-D on jejunal biopsy had both a higher number and higher activation of mucosal B lymphocytes and plasma cells [48]. This upregulation correlated positively with bowel movement frequency, stool form, and depression [48]. In a similar vein, the number of CD3^+^, CD8^+^, and CD25^+^ lymphocytes (as well as mast cells) has previously been found to be elevated in the colonic mucosa of a subgroup of those with IBS [49].

These lymphocytes secrete cytokines. Secretion of IL-1β from colonic CD3^+^/CD28^+^ T lymphocytes correlated modestly with bowel habit dissatisfaction in adults with IBS [50]. In adults with IBS induction of release of IL-6 (a pro-inflammatory cytokine) by pyridostigmine correlated with symptom exacerbation [51]. Cytokines also may have ties to psychosocial distress as adults with IBS-D who had increased anxiety, and depression scores were found to have higher TNF-α and IL-8 production in peripheral blood mononuclear cells [52]. Zhen et al. also found a correlation of these elevated cytokines with epithelial cell dysfunction as measured by occludin protein levels [52]. Whether larger numbers of lymphocytes or cytokines are found in children with IBS is currently unknown.

#### Innate immune activation

Defensins are antimicrobial peptides involved in the innate immune response [53]. They are stored in neutrophils, macrophages, and epithelial cells. These molecules are active against bacteria, fungi, and enveloped viruses [53]. A recent study in adults with IBS (versus healthy controls) found elevated human beta-defensin-2 levels in the stool, akin to that seen in patients with ulcerative colitis [54]. Langhorst et al. also found elevated beta-defensin-2 within the epithelial cells of those with IBS who had elevated fecal levels [54]. Whether similar findings are seen in children with IBS remains to be determined. Furthermore, therapies to directly bolster the innate immune system have not yet been conducted in subjects with IBS.

#### Enteroendocrine cells

Another area of neuroimmune activation in IBS involves enteroendocrine cells. The role of these cells in IBS recently has been extensively reviewed [55]. Briefly, enteroendocrine cells form part of the epithelial barrier from the stomach through the rectum. These cells are composed of different types dependent on location and their secreted neurohormonal mediators. These mediators include serotonin, somatostatin, ghrelin, polypeptide YY, cholecystokinin, motilin, gastric inhibitory peptide, pancreatic polypeptide, and enteroglucagon amongst others. Secretion of these mediators is activated by luminal stimuli such as nutrients and gut bacteria. These mediators are secreted into the lamina propria where they may have either local paracrine effects or, via blood, endocrine effects. Through their interaction with each other and the enteric, autonomic, and central nervous systems, these cells regulate several important gastrointestinal functions including visceral sensation, gut motility, intestinal secretion, intestinal absorption, nutrient uptake, and immunological defense [55].

Abnormalities in endocrine cell densities in the stomach, duodenum, ileum, colon, and rectum in adults with IBS have been found by investigators [55]. These abnormalities may vary by IBS subtype; for example, ghrelin-containing endocrine cells are higher in number per microscopic high-power field in adults with IBS-D but lower in adults with IBS-C [56]. In addition, certain chromogranins and secretogranins, which are proteins that can be secreted by enteroendocrine cells and serve as a markers for activity of the enteric neuroendocrine system, have been found to be elevated in adults with IBS [57]. Given both these cell density abnormalities and expression of protein mediators, it has been postulated that enteroendocrine cells may have a causative role in IBS. Similar findings have not been reported in children with IBS to date. In addition, there have not been any trials directed specifically at enteroendocrine cell function in IBS. Nevertheless, we anticipate that further work in this area will emerge over the next few years.

### Diet in IBS

Carbohydrate malabsorption as a pathway toward generation of symptoms in IBS has received a lot of recent attention, particularly in light of the efficacy of a low fermentable, oligosaccharide, disaccharide, monosaccharide, and polyols (FODMAP) diet in treating abdominal pain in IBS [58]. FODMAP carbohydrates include lactose, fructose, fructans, galactans, and polyols such as sorbitol. When ingested, they have both an intraluminal fermentation (gas-producing) and osmotic effect [59]. It is these physiologic changes which are believed to exacerbate IBS symptoms.

Diets which eliminate one substrate (e.g., lactose) at a time have not demonstrated efficacy in randomized controlled trials for childhood FGIDs [60]. However, elimination of a more comprehensive group appears to be more successful. A low FODMAP diet has demonstrated efficacy in ameliorating GI symptoms in children and adults with IBS in double blind, randomized controlled trials [61, 62]. In addition, low FODMAP diets alter the gut microbiome composition. One study found that a 4-week low FODMAP diet decreased luminal *Bifidobacteria* versus a habitual diet [63]. Another study in adults with IBS during a randomized, crossover, double blind trial found decreases in total bacterial abundance and diversity on the low FODMAP diet versus a typical Australian diet [62]. The role of these changes in the gut microbiome in affecting IBS symptoms while on the low FODMAP diet remains to be elucidated.

Lactose (one of the FODMAP carbohydrates) has received attention for many decades as a potential culprit for inducing gastrointestinal symptoms. Lactose malabsorption is not more prevalent in adults with IBS. Kumar et al. evaluated the prevalence of known lactase polymorphisms in those with IBS in comparison to healthy controls and found a similar frequency between groups [64]. However, underlying IBS pathological factors may play a role in lactose malabsorption symptom expression. Yang et al. evaluated lactose malabsorption in adults with IBS-D and healthy adults via a randomized blinded crossover trial; both groups had the lactase non-persistence polymorphism (C/C-13910). Subjects received doses of 10, 20, and 40 g of lactose [65]. In addition, they underwent hydrogen breath testing to identify lactose malabsorption, rectal barostat testing to measure visceral hypersensitivity, and ileocolonic biopsies to assess neuroimmune activation. Yang et al. found that those with an increase in gastrointestinal symptoms in response to lactose malabsorption had both increased visceral hypersensitivity and increased ileocolonic mast cells compared to both healthy controls and those with IBS who had no increase in GI symptoms following lactose malabsorption [65].

Dietary fiber supplementation, acting as a bulking agent, has long been proposed as a therapy for IBS. Based on meta-analyses and systematic reviews, the efficacy of fiber treatment in IBS is mixed; conclusions vary based on the different types of fiber used and, in part, on the different review methods employed [66–71]. Although not conclusive, data suggest that psyllium, a soluble fiber, reduces abdominal pain and/or improves stooling symptoms in adults with IBS [70–72]. A preliminary report suggests efficacy for psyllium fiber supplementation in children with IBS [73].

### Bile acids

The role of bile acids in gut physiology and potential role in FGIDs has recently been reviewed [74, 75]. Briefly, bile acids are steroid-derived detergent molecules produced within the liver and secreted into the intestinal lumen that aid in fat digestion. Primary bile acids (produced by the liver) include both chenodeoxycholic acid (CDCA) and cholic acid. In normal physiologic conditions ~95 % of secreted bile acids are absorbed in the ileum via the ileal bile acid transporter and become part of the enterohepatic circulation. Within the ileal enterocyte bile acids induce expression of nuclear farnesoid X receptor (FXR); this in turn leads to expression of fibroblast growth factor 19 (FGF-19). FGF-19 is secreted and activates fibroblast growth factor receptor 4 on the hepatocyte membrane; this is followed by downregulation of both cholesterol 7 alpha-hydroxylase and subsequent bile acid synthesis. Therefore, bile acids absorbed in the ileum normally have negative feedback on hepatic bile acid production.

Primary bile acids that reach the colon such as CDCA may have both potent colonic secretory and motor stimulatory effects [76]. However, the majority of bile acids that are not absorbed in the ileum are believed to undergo deconjugation and dihydroxylation by colonic microbiota, leading to the formation of secondary bile acids: deoxycholic acid and lithocholic acid. As a result, the potential effects of primary bile acids may be altered. For example, cholic acid does not have colonic secretory effects, whereas following its conversion to deoxycholic acid by colonic microbiota, it does [77].

Altered bile acid secretion/absorption and/or metabolism may affect gastrointestinal physiology in those with FGIDs. In a recent meta-analysis, bile salt malabsorption (measured via 23-seleno-25-homotaurocholic acid testing) was found in 28.1 % of adults with IBS-D [78]. Shin et al. demonstrated that adults with IBS-D placed on a high-fat diet (versus those with IBS-C) have higher amounts of both fecal fat and fecal unconjugated secretory bile acids [79]. Though not yet studied in children with IBS one study in children with functional constipation identified that a small subset had excessive sulfation of CDCA [80]. Given the secretory capability of CDCA and its loss following sulfation the authors hypothesized that this may contribute to constipation [80]. A small open-label study of the low FODMAP diet in children with IBS found those who had marked improvement (versus those who did not) while on the diet had higher amounts (amongst other metabolites) of fecal dehydroxylithocholate [9]. Future studies regarding bile acids and their subsequent physiologic effects in children with IBS are needed.

In open-label studies in adults with bile acid malabsorption, bile acid sequestrants such as colestipol have shown efficacy [81]. In addition, obeticholic acid (an FXR agonist) used in a small study of adults with IBS-D with bile acid diarrhea demonstrated both an increase in FGF19 and improvements in several clinical parameters including median stool frequency, stool form, and diarrhea [82]. Studies with these interventions have not been conducted in children with IBS to date.

CDCA has been found to have several beneficial effects in adults with IBS-C including acceleration of colonic transit, increased stool frequency, and loosening of stool consistency [83]. Using an ileal bile acid transporter inhibitor (thereby increasing bile acids in the colon) within a phase II trial for adults with chronic idiopathic constipation, Chey et al. demonstrated an improvement in both stool frequency and constipation-related symptoms [84]. We anticipate further studies attempting to increase colonic secretory bile acid effects in subjects (both adults and children) with IBS-C in the future.

### Gut microbiome

The gut microbiome (collection of microbiota and their genetic material) and its role in health and disease are being increasingly recognized. The gut microbiome composition of adults and children with IBS (versus healthy controls) has been found to be different [85, 86]. Using both 16s rRNA pyrosequencing and PhyloChip technology on fecal samples from children with IBS (versus healthy controls), Saulnier et al. found a significantly greater percentage of the class *Gammaproteobacteria* (such as *H. parainfluenzae*) as well as a novel Ruminococcus-like microbe [86]. In addition, Saulnier et al. found that in children with IBS, microbiome composition correlated with both abdominal pain severity and frequency; moreover, microbiome composition was also able to differentiate those with an IBS-U versus IBS-C subtype. Gut microbiome composition was potentially found to be important as a biomarker for improvement on a low FODMAP diet; children who markedly improved had a different microbiome composition as compared to those who did not [9, 61].

The gut microbiome has several functions in health including fermentation/degradation of undigested proteins and carbohydrates, hydrogen disposal, and bile acid transformation [87]. When performing these functions, the microbiome produces several metabolites including but not limited to short-chain fatty acids; gases, such as hydrogen sulfide; and secondary bile acids derived from primary bile acids. Through their presence and metabolic function, gut microbiota may play a role in several of the pathological factors covered so far in this review including dysregulated intestinal immune function, chronic low-grade mucosal inflammation, increased gut permeability, psychosocial distress, and visceral hypersensitivity [41, 87, 88]. Further work to elucidate the mechanisms of action of the gut microbiome and/or their metabolites within the biopsychosocial model of IBS (Fig. 1) is needed.

Therapies which are directed toward the microbiome such as probiotics have demonstrated efficacy in children with IBS. A recent meta-analysis of *Lactobacillus rhamnosus* GG in children with IBS found a significantly higher rate of treatment responders (no pain or improvement in pain) in those receiving the probiotic versus placebo [89]. In a multicenter, randomized, placebo-controlled, double blind, crossover study in children with IBS, VSL#3 significantly ameliorated gastrointestinal symptoms [90]. The mechanisms involved with these improvements through the use of probiotics remain largely to be elucidated.

### Summary

Children with IBS may have several interacting factors which play a role in the pathogenesis of their symptoms. These factors are being actively investigated and, given their interrelated nature, are better understood when placed within the context of the biopsychosocial model (Fig. 1). Underpinning these factors are cellular and molecular mechanisms. Future investigations of these mechanisms can be challenging given the likely heterogeneous etiologies driving the symptoms of IBS. Ideally, mechanisms should not be investigated in isolation; rather, interrelationships can be investigated/accounted for within the biopsychosocial model. These mechanisms, when elucidated further, may lead to identification of biomarkers and personalized therapies for children with IBS.

## Abbreviations

5-HT: 5-hydoxytryptamine
5-HT3: 5-hydroxytryptamine receptor 3
5-HTTLPR: 5-HT transporter-linked polymorphic region
CDCA: chenodeoxycholic acid
CGRP: calcitonin-related gene peptide
FGID: functional gastrointestinal disorder
FGF19: fibroblast growth factor 19
FODMAP: fermentable oligosaccharides, disaccharides, monosaccharides, and polyols
FXR: farnesoid X receptor
H1R: histamine-1 receptor
IBS: irritable bowel syndrome
IBS-C: IBS with constipation
IBS-D: IBS with diarrhea
IBS-M: IBS mixed subtype
IBS-U: IBS un-subtyped
Ig: immunoglobulins
NGF: neuronal growth factor
NK1: neurokinin 1 receptor
PAR2: proteinase-activated receptor-2
PG: prostaglandins
SERT: serotonin transporter
SP: substance P
TLR: toll-like receptor
TrkA: receptor for nerve growth factor
TRPV1: transient receptor vanilloid 1
